# Effects of Charge Density on Photophysics and Aggregation Behavior of Anionic Fluorene-Arylene Conjugated Polyelectrolytes

**DOI:** 10.3390/polym10030258

**Published:** 2018-03-02

**Authors:** Liliana M. Martelo, Sofia M. Fonseca, Ana T. Marques, Hugh D. Burrows, Artur J. M. Valente, Licínia L. G. Justino, Ullrich Scherf, Swapna Pradhan, Qiu Song

**Affiliations:** 1 Departamento de Química, Universidade de Coimbra, 3004-535 Coimbra, Portugal; sfonseca@qui.uc.pt (S.M.F.); avalente@ci.uc.pt (A.J.M.V.); liciniaj@ci.uc.pt (L.L.G.J.); 2 Makromolekulare Chemie, Bergische Universität Wuppertal, DE-42097 Wuppertal, Germany; anatmarques@yahoo.com (A.T.M.); scherf@uni-wuppertal.de (U.S.); SPradhan@alert.com (S.P.); squi2010@sinano.ac.cn (Q.S.)

**Keywords:** conjugated polyelectrolytes, aggregation, fluorene-phenylene copolymers, photophysics

## Abstract

Three anionic fluorene-based alternating conjugated polyelectrolytes (CPEs) have been synthesized that have 9,9-bis(4-phenoxy-butylsulfonate) fluorene-2,7-diyl and 1,4-phenylene (PBS-PFP), 4,4′-biphenylene (PBS-PFP2), or 4,4″-*p*-terphenylene (PBS-PFP3) groups, and the effect of the length of the oligophenylene spacer on their aggregation and photophysics has been studied. All form metastable dispersions in water, but can be solubilized using methanol, acetonitrile, or dioxane as cosolvents. This leads to increases in their emission intensities and blue shifts in fluorescence maxima due to break-up of aggregates. In addition, the emission maximum shifts to the blue and the loss of vibronic structure are observed when the number of phenylene rings is increased. Debsity Functional Theory (DFT) calculations suggest that this is due to increasing conformational flexibility as the number of phenylene rings increases. This is supported by increasing amplitude in the fast component in the fluorescence decay. The nonionic surfactant *n*-dodecylpentaoxyethylene glycol ether (C_12_E_5_) also breaks up aggregates, as seen by changes in fluorescence intensity and maximum. However, the loss in vibrational structure is less pronounced in this case, possibly due to a more rigid environment in the mixed surfactant-CPE aggregates. Further information on the aggregates formed with C_12_E_5_ was obtained by electrical conductivity measurements, which showed an initial increase in specific conductivity upon addition of surfactants, while at higher surfactant/CPE molar ratios a plateau was observed. The specific conductance in the plateau region decreased in the order PBS-PFP3 < PBS-PFP2 < PBS-PFP, in agreement with the change in charge density on the CPE. The reverse process of aggregate formation has been studied by injecting small volumes of solutions of CPEs dissolved at the molecular level in a good solvent system (50% methanol-water) into the poor solvent, water. Aggregation was monitored by changes in both fluorescence and light scattering. The rate of aggregation increases with hydrophobicity and concentration of sodium chloride but is only weakly dependent on temperature.

## 1. Introduction

Conjugated polyelectrolytes (CPEs) have emerged as advanced materials, which combine the electronic, spectroscopic, and photophysical properties of conjugated organic polymers with the solubility in water and polar solvents of ionic compounds [1,2,3]. Their aqueous solubility makes them valuable as sensors of biological or chemical systems [4,5,6,7,8,9], whilst their ionic character provides the potential for use in optoelectronics [10], as charge injection or transport layers [11], in light emitting devices [12], or as solar concentrators [13]. In addition, as polyelectrolytes, they can self-assemble with oppositely charged species, such as surfactants, to build up complex multilayer structures with various potential materials applications [14,15]. They can also bind oppositely charged electronic energy acceptors, such as [Ru(bpy)_3_]^2+^, for artificial light harvesting [16]. Fluorene-based copolymers are particularly attractive for many of these applications because of their excellent chemical and photophysical properties [17,18].

However, frequently CPEs are not present as isolated polymer chains, and they tend to aggregate in water [6,19,20,21,22,23,24,25,26,27] and other specific solvents, such as methanol [28]. This reduces both solubility and fluorescence quantum yields, frequently leading to spectral shifts. This is a particularly serious problem with rigid rod polyelectrolytes, such as those that have a poly(*p*-phenylene) backbone, as well as the closely related fluorene-based polymers [22,23,24,25,26,27,28]. For example, stirring the anionic alternating copolymer poly{[9,9-bis(4-phenoxybutylsulfonate)]fluorene-2,7-diyl-*alt*-1,4-phenylene} (PBS-PFP) in water leads to the formation of a metastable dispersion of polymer clusters [24,26]. These can be broken up to give a thermodynamically stable solution by adding co-solvents [29], or non-ionic surfactants, such as *n*-dodecylpentaoxyethylene glycol ether (C_12_E_5_) [24,26,30,31]. In the case of surfactants, this occurs in the region of the critical micelle concentration (cmc) [24,30,32]. Various factors appear to be involved in the formation and break-up of CPE aggregates [28,29,30,31,33,34], although, as of yet, there is no consensus as to the dominant interaction(s). Edman and co-workers have demonstrated, using pulse-field-gradient Nuclear magnetic resonance (NMR,) photoluminescence and Raman spectroscopy, that the CPE poly{9,9′-bis[6″-(*N*,*N*,*N*-trimethylammonium)-hexylfluorene-*alt*-1,4-phenylene] dibromide} forms aggregates in methanol [28]; they have also suggested that π,π interactions of backbones, coupled with electrostatic screening of ionic side chains by solvent, are important effects. Molecular dynamics simulations of the aggregation of PBS-PFP tetramers in water suggest that both electrostatic and hydrophobic interactions are involved in the association [29]. This is supported by small angle X-ray and neutron scattering studies on aqueous solutions of the anionic poly[2,2′-bithiophene-*alt*-9,9-bis(4-sulfonylbutoxyphenyl)fluorene-2,7-diyl], coupled with simulated ab initio annealing, which suggest loose, preferentially two-dimensional aggregates [35].

**Figure 1:**
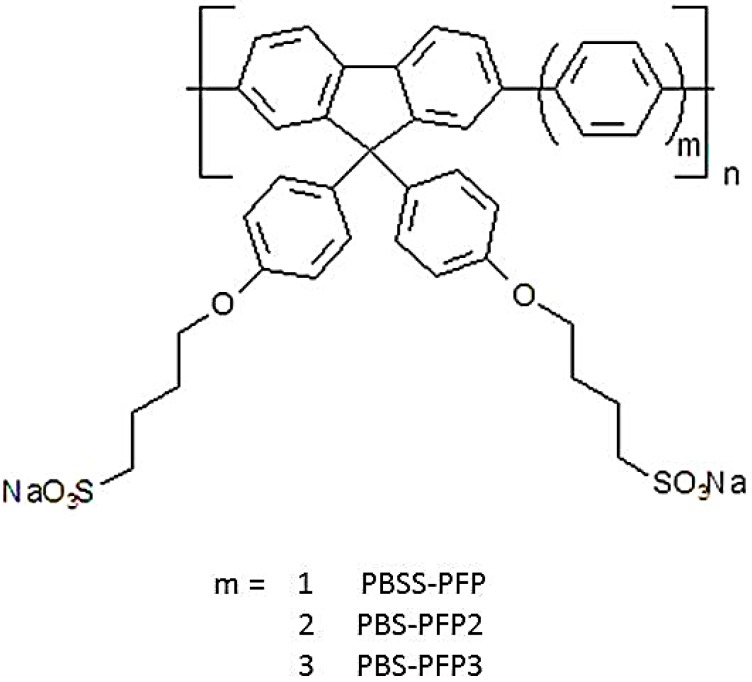
Structures of the fluorene-arylene conjugated polyelectrolytes studied.

To obtain more insight into the effect of decreasing charge density and increasing hydrophobicity on the aggregation behavior, we have carried out a comparative study of the three anionic, fluorene-arylene conjugated polyelectrolytes of increasing hydrophobicity shown in Figure 1, which have, respectively, 1,4-phenylene (PBS-PFP), 4,4′-biphenylene (PBS-PFP2), and 4,4″-*p*-terphenylene (PBS-PFP3) spacer groups. Increasing the spacer length between the anionic groups decreases the charge density, which may also affect the aggregation. 

Although many experimental studies [28,29,30,31,33,34,35,36] have provided strong evidence for aggregation, and this is supported by molecular dynamics simulations of the association of two initially isolated oligomers [29], we have not found any reports regarding the kinetics of this process. This is surprising, as this would be expected to provide indications of the driving force(s) of the aggregation. In this paper we also report an experimental study of the kinetics of aggregation for this family of anionic CPEs.

## 2. Materials and Methods

### 2.1. Materials

The synthesis of poly{[9,9-bis(4-phenoxybutylsulfonate)]fluorene-2,7-diyl-*alt*-1,4-phenylene}, (PBS-PFP, *M*_n_: 6500) has been described in detail elsewhere [24]. The two homologous conjugated polyelectrolytes poly{[9,9-bis(4-phenoxybutylsulfonate)]fluorene-2,7-diyl-*alt*-4,4′-biphenylene} (PBS-PFP2) and poly{[9,9-bis(4-phenoxybutylsulfonate)]fluorene-2,7-diyl-*alt*-4,4″-*p*-terphenylene} (PBS-PFP3) were synthesised by a similar Suzuki-type cross coupling to that described for PBS-PFP with *M*_n_ values of 4000–6000 Gel permeation chromatography (GPC) after dialysis in water using a dialysis membrane cutoff of 3500 g·mol^−1^. Organic solvents were of the purest grade available and were used as received. All experiments were carried out in solutions in Milli-Q Millipore water. The concentrations of the polyelectrolyte solutions (in terms of repeat units, RU) were determined using the molecular weights of the respective RUs. For kinetic studies on aggregation, the CPEs were dissolved at the molecular level in 50% methanol-water (a good solvent system), and small volumes (0.3 mL) of this solution were injected into water (2.7 mL). For this, the solutions of the polyelectrolytes were freshly prepared in 50% methanol-water and were stirred overnight before use. 

### 2.2. Instrumentation and Methods

Absorption and luminescence spectra were recorded on Shimadzu UV-2100 (Kyoto, Japan) and Horiba-Jobin-Ivon SPEX Fluorolog 3-22 spectrometers (Kyoto, Japan), respectively. Fluorescence spectra were corrected for the wavelength response of the system. When not being used for measurements, all samples were kept in the absence of light. Light scattering experiments were performed on the polyelectrolyte solutions with the same spectrofluorimeter using the method described by Mougán and co-workers [37], with excitation and observation at 500 nm (slit width 0.5 mm). The system used for electrical conductivity measurements has been described in detail elsewhere [30,36]. The cell constant (approximately 0.1012 cm^−1^) was determined using the procedure and data from Barthel et al. [38]. 

The geometries of molecular models of PBS-PFP, PBS-PFP2, and PBS-PFP3 were optimized at the DFT level without symmetry constraints using the GAMESS (US, maintained by members of the Gordon Research Group, Iowa State University), [39] code. The calculations employed the B3LYP functional, which combines the hybrid exchange functional of Becke [40] with the correlation functional of Lee, Yang, and Parr [41] (LYP) and the 6-311G(d,p) basis sets for the expansion of the Kohn-Sham orbitals. The gradient threshold for optimizations was taken as 10^−5^ hartree bohr^−1^.

Time-resolved fluorescence decays were performed using a home-built picosecond Time correlated single photon counting (TCSPC) apparatus, described in detail elsewhere [42]. Excitation was at 392 nm. Fluorescence decays and the instrumental response function (IRF) were collected using 4096 channels in a 0.814 ps/channel scale until 5 × 103 counts at maximum were reached. The full width at half-maximum (fwhw) of the IRF was about 22 ps and was highly reproducible. The deconvolution of the fluorescence decays was made with modulating functions method of Striker [43]. 

## 3. Results

As with our previous studies with PBS-PFP [24,26,29,30], in all cases, the CPEs formed metastable dispersions in water but could be dissolved at the molecular level upon addition of the organic co-solvents methanol, acetonitrile, or dioxane, or the non-ionic surfactant C_12_E_5_. This can be seen clearly through changes in their fluorescence spectra. The spectra in water were similar for all three CPEs (Supporting information Figure S1). There was an approximately five-fold increase in fluorescence intensity and a blue shift in the emission band positions upon addition of co-solvent or C_12_E_5_ above its *cmc*, leading to relatively high fluorescence quantum yields, as shown in the Table 1. Data are given for 50% methanol-water, but similar results are obtained with acetonitrile-water and dioxane-water binary solvent mixtures.

**Figure 2:**
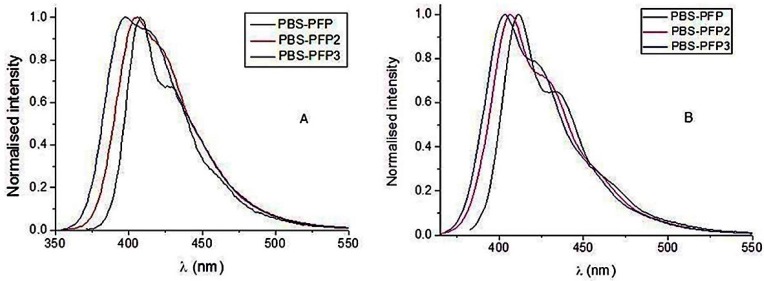
(**A**) Normalized fluorescence spectra in 50% acetonitrile-water for solution of PBS-PFP (6.32 × 10^−5^ M in terms of repeat unit) (black line), PBS-PFP2 (5.69 × 10^−5^ M in terms of repeat unit) (red line), and PBS-PFP3 (4.54 × 10^−5^ M in terms of repeat unit) (blue line). The excitation wavelength was 370 nm. (**B**) Normalized fluorescence spectra in water with the presence of the surfactant C_12_E_5_ solution (1 × 10^−4^ M) of PBS-PFP (5.54 × 10^−5^ M in terms of repeat unit) (black line), PBS-PFP2 (6.07 × 10^−5^ M in terms of repeat unit) (red line), and PBS-PFP3 (5.55 × 10^−5^ M in terms of repeat unit) (blue line). The excitation wavelength was 370 nm.

The amount of co-solvent needed to break up the clusters was found to increase in the order PBS-PFP < PBS-PFP2 < PBS-PFP3, reflecting increasing hydrophobicity and decreasing charge density on these hairy rod-type copolymers. For example, although ≈20% dioxane was sufficient to induce increases in fluorescence intensity with PBS-PFP [29], indicating de-aggregation, with PBS-PFP2, ≈40% dioxane was necessary. However, the actual dependence on the mole fraction of co-solvent was complex, possibly as a result of the importance of selective solvation in these systems [29]. The bands in the fluorescence spectra all shifted to shorter wavelengths upon addition of co-solvent or surfactant, in agreement with the break-up of aggregates being an important factor in the solubilization [29,30]. However, the extent of this blue shift depends on the number of arylene rings, as seen by changes in the first emission maximum in 50% acetonitrile-water solutions, PBS-PFP (407 nm) > PBS-PFP2 (405 nm) > PBS-PFP3 (398 nm), and illustrated in Figure 2A. Similar behavior is observed in the presence of C_12_E_5_, although the emission wavelengths are somewhat longer (PBS-PFP (415 nm) > PBS-PFP2 (408 nm) > PBS-PFP3 (402 nm), and Figure 2B), possibly suggesting a very slight increase in conjugation in the surfactant system compared with 50% acetonitrile-water.

**Figure 3:**
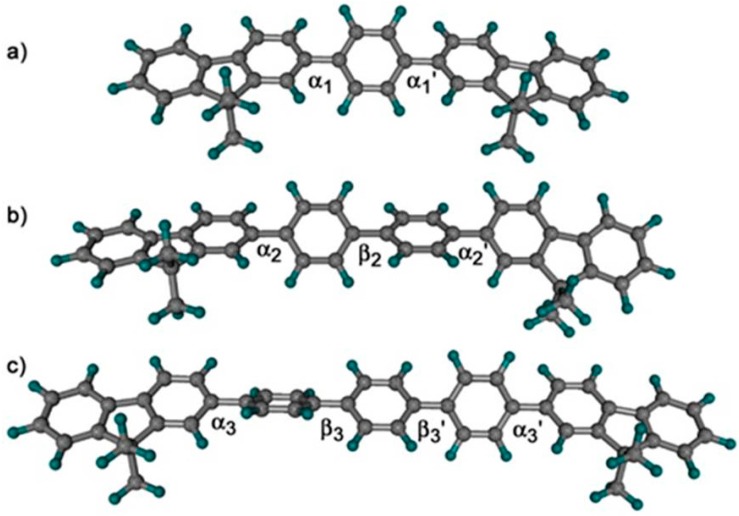
B3LYP/6311-G(d,p) optimized geometries of structural models of (**a**) PBS-PFP; (**b**) PBS-PFP2; and (**c**) PBS-PFP3. The phenoxybutylsulfonate chains at position 9 of the five-membered rings were replaced by methyl groups to reduce the computational time, in a similar manner to our procedure in ref. [44]. It has been shown that this does not significantly affect the equilibrium geometries [45].

This blue shift in the absorption and emission maxima in the PBS-PFPm series with an increasing number (m) of hydrophobic phenylene rings of the oligophenyl unit (Figure 2A,B) is accompanied by a decrease in the vibrational structure in the spectra. Both of these observations can readily be explained by the greater conformational flexibility obtained upon increasing the number of arylene rings. This will both decrease the conjugation length, shifting the emission to the blue, and broaden the emission band through reducing the rigidity. The decrease in vibronic structure was less pronounced in the C_12_E_5_ systems, suggesting a more rigid environment in the mixed CPE-surfactant aggregates. The effect of number of arylene units on the spacer length is illustrated by the structures of the three (9,9-dimethylfluorene)-4,4″-arylene-(9,9-dimethylfluorene) oligomers obtained by DFT calculations (Figure 3). In fact, for PBS-PFP, the inter-ring angles α1 and α1’ are, respectively, 37.59 and 37.58 degrees. For PBS-PFP2, the angles α2, β2, and α2’ are 38.37, 38.63, and 38.13 degrees, respectively, and for PBS-PFP3, α3, β3, β3’, and α3’ are 38.70, 39.81, 40.01, and 39.44. These results clearly show that the inter-unit torsion angles become larger as the number of arylene units increases. Additionally, we can see that the angles between two successive arylene units are larger than those between one fluorene and one arylene unit.

More detailed theoretical results on conformational flexibility of fluorene-phenylene copolymers have been reported elsewhere [46]. 

**Figure 4:**
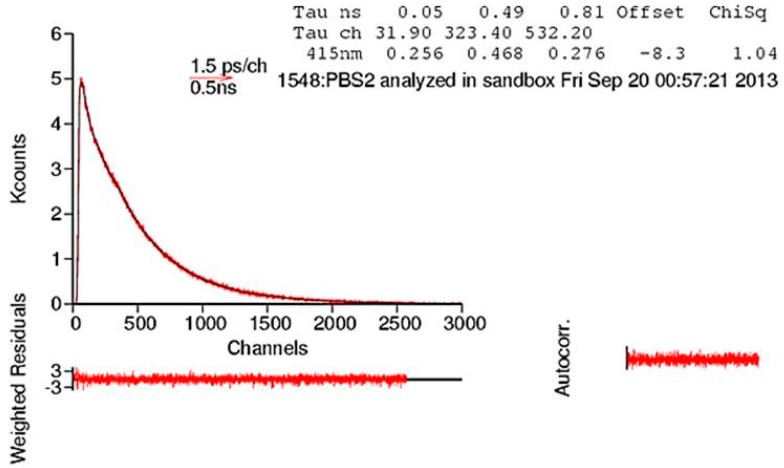
Fluorescence emission decay of PBS-PFP2 at 415 nm in a 1:1 MeOH/H_2_O solution fitted to the sum of three exponentials. Weighted residuals (W.R., scale −3 ≤ σ ≤ +3) and autocorrelation functions are also presented to show the quality of the fit.

To obtain further information on the greater conformational flexibility obtained upon increasing the number of arylene rings, we have also studied the fluorescence lifetimes of the CPE emission. The decays of the fluorescence of the three CPEs were studied in a 1:1 MeOH/H_2_O mixture. The sums of three discrete exponential functions were needed to fit the fluorescence decays of these three oligomers in 1:1 methanol-water. This contrasts with previous studies with PBS-PFP in 1:1 dioxane-water, in which the fluorescence decays could be fitted with two exponentials [47]. The reasons for this difference are not clear. However, in a related cationic fluorene-phenylene copolymer [48], a similar intermediate lifetime component (τ_2_) was observed and associated with residual CPE aggregation. It is known that the contribution of this component is strongly dependent on solvent composition and concentration [49], which may explain the difference between the two solvent media. The lifetimes and amplitudes of the three components are presented in Table 2 and a representative decay is shown in Figure 4. The long-decay component (τ_1_) for the three CPEs is associated with the natural fluorescence decay of the polymer. The lifetime for PBS-PFP is slightly longer than that reported for the free PBS-PFP in C_12_E_5_ micelles [24], or 1:1 dioxane-water [47]. The behavior of the shortest lifetime component (τ_3_), with a lifetime of tens of ps, is of particular importance for this study. This is attributed, at least in part, to conformational relaxation of the conjugated polymer backbone [50,51]. The excited singlet state decay of polyfluorenes depends on the molecular weight of the polymer [52], while the relative amplitude reflects the degree of conformational disordering. The lifetime of this short decay component only varies slightly on going from PBS-PFP to PBS-PFP3, which is in agreement with the three CPEs having similar molecular weights. However, the pre-exponential amplitude, a_3_, increases with an increase in the number of arylene rings, strongly suggesting an increase in the conformational flexibility of the polymer backbone in the order PBS-PFP < PBS-PFP2 < PBS-PFP3. This is in complete agreement with both the results of DFT calculations and with the suggestions from the blue shift in the emission maxima and the loss of vibronic structure in the fluorescence spectra. 

**Figure 5:**
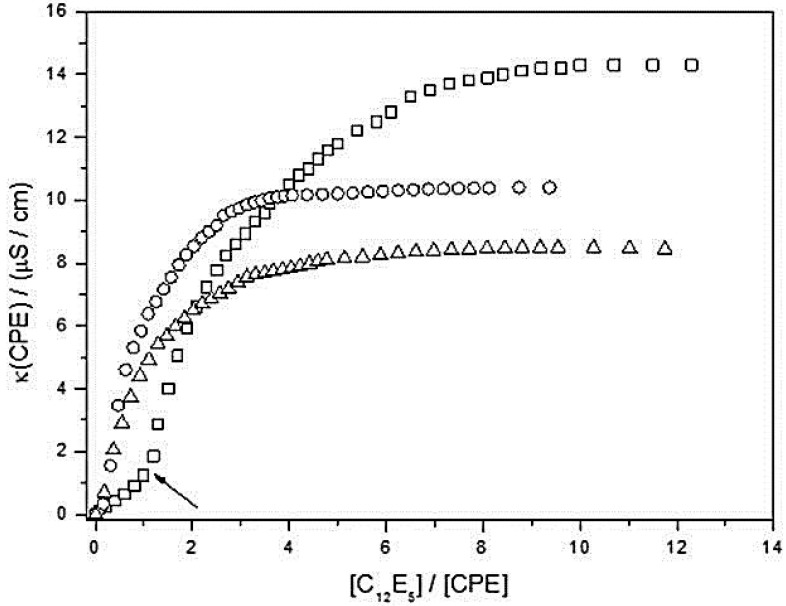
Changes in specific electrical conductance (κ) of aqueous solutions of CPEs upon adding C12E5, at 25.0 °C. (□) PBS-PFP, (o) PBS-PFP2, and (∆) PBS-PFP3. The arrow highlights the *cac* for PBS-PFP:C_12_E_15_ mixed solutions.

Further information on the solubilisation process was obtained from electrical conductivity measurements of the three CPEs in water in the presence of increasing concentrations of C_12_E_5_ (Figure 5). In all cases, an initial increase in specific conductivity was observed upon adding the non-ionic surfactant, while at higher surfactant/CPE molar ratios a plateau was observed. The specific conductance in the plateau region decreased in the order PBS-PFP (14.5 ± 0.1 μS/cm) > PBS-PFP2 (10.29 ± 0.05 μS/cm) > PBS-PFP3 (8.27 ± 0.05 μS/cm), which was in agreement with the decrease in charge density on the CPE. With PBS-PFP in aqueous C_12_E_5_ solution above the surfactant *cmc* (ca. 5 × 10^−5^ M) [24,53], it is strongly suggested from dynamic light scattering, SAXS and SANS scattering, and molecular dynamics simulations [26,30] that mixed cylindrical CPE-surfactant aggregates are formed. Electrical conductivity also provides information on the maximum concentration of C_12_E_5_ needed for the complete incorporation of CPE; thus, for the PBS-PFP, the plateau starts at molar ratio, MR = [C_12_E_5_]/[PBS-PFP], equal to 3.7 (±0.2); for PBS-PFP2 and PBS-PFP3, the corresponding computed values are: 1.3 (±0.1) and 1.69 (±0.04), respectively. These values were calculated by using the intersection of two straight lines of κ = *f*(MR), by using data for low and high MR values, and the experimental uncertainty is likely to be greater than the above standard deviations. However, it seems likely that 2 (±1) surfactants are associated with each conjugated polymer repeat unit. This value is similar to what is found in molecular dynamics simulations of PBS-PFP with C_12_E_5_ [30]. With the exception of the most soluble CPE, the complete solubilisation of PBS-PFP-2 and PBS-PFP-3 occurs for a mixture of 1:1 and 1:2 CPE:C_12_E_5_ stoichiometries. We believe that similar structures are formed in all three cases. Interestingly, from the conductivity data we can see a further inflexion point on the curves at low MR, illustrated in the Figure with an arrow for PBS-PFP, which can be assigned to a critical aggregation concentration (*cac*). The *cac* is the concentration needed to induce surfactant binding to the polyelectrolyte [54] and is more commonly associated with flexible polyelectrolytes [55] than with the more rigid systems in this study. In our case, the *cac* decreases with increasing hydrophobicity of the CPE (*cac* = 1.23 × 10^−4^ and 2.42 × 10^−5^ M for 1.25 × 10^−4^ M PBS-PFP and 1.55 × 10^−4^ M PBS-PFP2, respectively) and is barely perceptible with PBS-PFP3. The fact that no *cac* is observed for the PBS-PFP3 might be related to its high hydrophobicity. In the presence of this CPE all C_12_E_5_ is used to break-up the polymer clusters, and there is no significant concentration of “free” polymer chains. However, with the less hydrophobic (and more soluble) CPEs, it is possible that there will be in solution aggregates and “free” polymeric chains that will also be available to interact with the surfactant; consequently, the inflexion point at MR = 1.0 (±0.2) is readily observed for the least hydrophobic system, PBS-PFP (see arrow in Figure 5). This also justifies the higher MR, at which the maximum electrical conductance is reached with PBS-PFP/C_12_E_5_ solutions. This strongly suggests that we are not simply observing dissolution of the polymers in surfactant micelles but instead, with the relatively small CPEs in this study (around 10 repeat units), we are forming mixed polymer-surfactant structures. Molecular dynamics and SAXS results on the system of PBS-PFP with C_12_E_5_ are in complete agreement with this model [30]. Cryo-TEM studies of the PBS-PFP/C_12_E_5_ system suggest worm-like structures both with pure surfactant and mixed aggregates [30].

**Figure 6:**
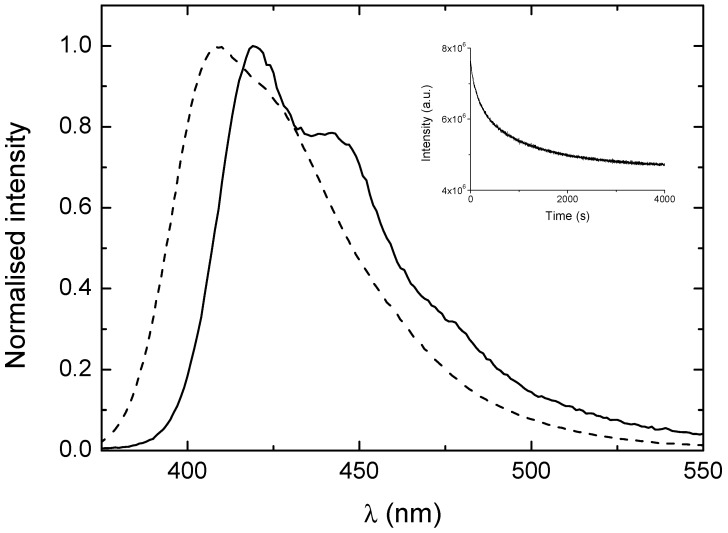
Fluorescence spectra of PBS-PFP3 in water (solid line) and 50% methanol-water (dashed line) solutions. Inset: Changes in fluorescence intensity resulting from aggregation on injecting a solution of PBS-PFP3 (in 50% methanol-water) into water.

While much of the work has focused on the breaking up of these CPE aggregates, we have also looked at the reverse process and the formation of aggregates. This was followed by injecting small volumes (≤10%) of the CPE solutions in 50% methanol-water into pure water and observing the resulting changes in fluorescence. Typical data for PBS-PFP3 is shown in the inset of Figure 6. As can be seen, the aggregation process is relatively slow (t½ ≈ 10 min). The timescale for the aggregation process is close to that observed for aggregation and beta-phase formation of the hairy-rod poly(9,9-di-*n*-alkylfluorene)s in the poor solvent methylcyclohexane [56,57], in which the driving force appears to involve largely lipophilic (hydrophobic) interactions. 

**Figure 7:**
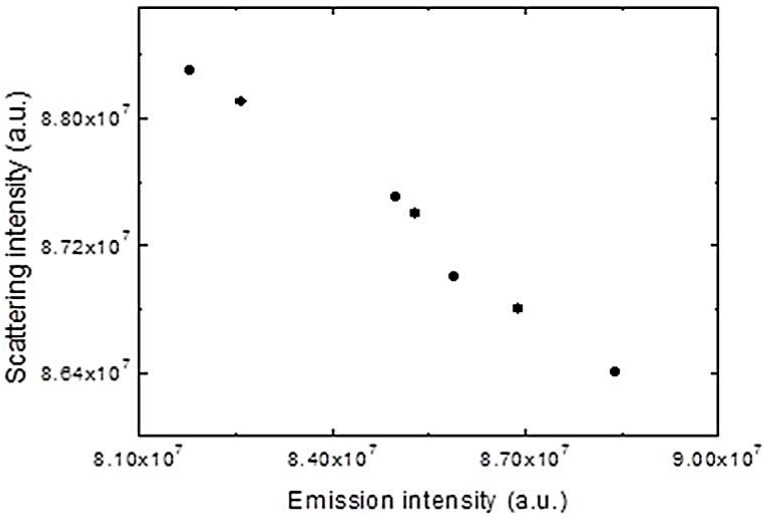
Plot of light scattering at 500 nm against fluorescence intensity at the emission maximum (415 nm) upon injecting a solution of PBS-PFP3 in 50% methanol-water into pure water.

Confirmation that we are dealing with an aggregation process is obtained by light scattering experiments. The fluorescence changes upon the injection of solutions of the CPEs in 50% methanol-water into pure water are accompanied by increases in turbidity, as can be seen in Figure 7, in which the fluorescence intensity at the emission maximum is proportional to the light scattering for the same observation times, and both are attributed to aggregation, which increases with time. 

The aggregation (see inset in Figure 6) followed complex kinetic behavior, which could not be fitted over the whole time range by any simple kinetic expression. However, the initial decay can be treated as a single exponential, and as we are more interested in understanding the factors that influence the aggregation than in obtaining a detailed analytical fit to the kinetic behavior, we will assume that it can be considered as a pseudo-first-order reaction. Since we are not following the decay over the whole reaction, we need a method to estimate the final emission intensity. One commonly used technique for first-order rate constant evaluation, in the absence of a final reading, is Guggenheim’s method [58,59]. We have determined the pseudo-first-order rate constants for the aggregation process of the three polyelectrolytes under study, and data is presented in Table 3. For PBS-PFP2 we also investigated the effect of adding salt by injecting the polyelectrolyte solutions in pure water and in aqueous solutions with various concentrations of NaCl. In addition, the effect of temperature on the aggregation process was studied with this CPE. The effect of polyelectrolyte concentration on the rate constants was investigated for PBS-PFP3. 

The values for the series PBS-PFP (0.90 × 10^−3^ s^−1^), PBS-PFP2 (1.4 × 10^−3^ s^−1^), and PBS-PFP3 (2.0 × 10^−3^ s^−1^) under similar conditions show an increase in the pseudo-first-order rate constants for the aggregation process with increasing hydrophobicity and decreasing charge density. This is in agreement with the above suggestion that lipophilic interactions may be important. Molecular dynamics simulations of the aggregation of two PBS-PFP oligomers in aqueous solution suggest that both hydrophobic and electrostatic interactions are involved in the association/dissociation process of aggregate formation [29]. With PBS-PFP2, an increase in the pseudo-first-order rate constants for the aggregation process is observed upon the increase of the salt concentration of the solution. Since salt is expected to screen the Coulombic interactions, this suggests that increasing the sodium ion concentration leads to a decrease in the electrostatic repulsions between the anionic sulfonate groups of CPE chains, thus favouring the aggregation process. It is worth noting that salt also decreases the cloud point in mixtures of PBS-PFP and non-ionic alkyloxyethylene surfactants in water [34], again indicating the importance of electrostatic interactions. The effect of temperature on the aggregation rate constants is less pronounced, with only a small increase in the rate constant (activation energy ≤ 14.7 kJ·mol^−1^) being observed on increasing the temperature. The activation energy is very close to that of self-diffusion in water (15 kJ·mol^−1^) [60], suggesting a role of diffusion and water viscosity in both formation and break-up of aggregate species. 

The effect of varying the polyelectrolyte concentration upon the aggregation was studied with PBS-PFP3. The rate constants obtained were very similar, which may reflect both the limitations of fitting the kinetic behavior to a pseudo-first-order process and the importance of homogeneous (spontaneous) nucleation [61] in the aggregation. This parallels the behavior seen with formation of poly(9,9-di-*n*-alkylfluorene) β-phase in the poor solvent methylcyclohexane [56].

## 4. Conclusions

The aggregation behavior in water has been studied in three alternating copolymers that have 9,9-bis(4-phenoxy-butylsulfonate)fluorene-2,7-diyl and 1,4-phenylene (PBS-PFP), 4,4′-biphenylene (PBS-PFP2), or 4,4″-*p*-terphenylene (PBS-PFP3) groups. These can be solubilized with the organic cosolvents methanol, acetonitrile, or dioxane, with the amount of cosolvent that is necessary increasing with the increasing hydrophobicity of the CPE (unpublished observations, not shown in detail). In contrast, when the non-ionic surfactant C_12_E_5_ is used to dissolve these copolymers at the molecular level, electrical conductivity studies show that the amount needed decreases with the increasing hydrophobicity of the CPE, due to formation of mixed surfactant-polymer aggregates rather than simple dissolution in micelles. The effects of hydrophobicity and charge density of the CPE on the polymer/surfactant interactions are in agreement with the discussions of Guzmán et al. [62]. Fluorescence spectra and lifetimes, coupled with DFT calculations, indicate that the backbone conformational flexibility increases with the number of phenylene rings (PBS-PFP3 > PBS-PFP2 > PBS-PFP). However, fluorescence spectra suggest a more rigid environment for the CPEs in the aggregates with C_12_E_5_ than in mixed aqueous/organic solvents. 

In previous work with PBS-PFP [24,26,29,30], we have obtained information on aggregation in aqueous solution and of the effects of cosolvent and surfactants on breaking up these aggregates. The present study has been designed to study the effects of increasing hydrophobicity on these by increasing the number of hydrophobic 1,4-phenylene rings between fluorene units in alternating copolymers. As well as the general insights obtained, two important observations are relevant for future applications of these CPEs. Firstly, the marked blue shift in fluorescence on going from PBS-PFP to PBS-PFP2 and PBS-PFP3 provides color tuning for blue-conjugated, polymer-based light emitting diodes PLEDS, which is likely to be important in the quest for commercially viable organic white LEDS [63]. Secondly, increasing the number of phenylene rings in these CPEs may favour interchain π–π interactions, which are relevant for applications in charge transport layers. Future experiments should be designed to test these ideas in devices. 

The reverse process of CPE aggregation has been studied. The rates of aggregation are relatively slow, with low activation energies, suggesting relatively weak interchain interactions. This is in agreement with the rather loose aggregates suggested from SANS and SAXS data for a closely related anionic CPE in water [35]. Aggregation in colloidal systems frequently involves a balance between different interactions [64,65], and from the effects of added salt and of the hydrophobicity of the CPE chain on the rate, we believe that the results are best interpreted as resulting from competition between electrostatic repulsion between the CPE chains and a much weaker attractive interaction; this most likely involves localized π–π and other lipophilic interactions between the polymer backbones and electrostatic attraction between CPE sulfonate groups and counterions. This is in agreement with results from the molecular dynamics simulations [29]. We believe that similar considerations may be important in the aggregation of other CPEs in an aqueous solution.

## Figures and Tables

**Table 1:** Fluorescence quantum yields for the CPEs in study determined in co-solvent solution and using the non-ionic surfactant C_12_E_5_ above its *cmc* in aqueous solution (50 μM).

CPEs	$\phi_{F}$ C_12_E_5_/H_2_O	$\phi_{F}$ 1:1 MeOH/H_2_0
PBS-PFP	0.60	0.59
PBS-PFP2	0.66	0.64
PBS-PFP3	0.61	0.60

**Table 2:** Decay times and amplitudes in a 1:1 MeOH/H_2_O solution of PBS-PFP (6.84 × 10^−5^ M in terms of repeat unit), PBS-PFP2 (6.43 × 10^−5^ M in terms of repeat unit), and PBS-PFP3 (5.56 × 10^−5^ M in terms of repeat unit).

	τ_1_ (ns)	τ_2_ (ns)	τ_3_ (ns)	a_1_	a_2_	a_3_
PBS-PFP	1.80	0.66	0.05	0.328	0.625	0.046
PBS-PFP2	0.80	0.49	0.05	0.291	0.454	0.255
PBS-PFP3	1.26	0.53	0.08	0.305	0.388	0.307

**Table 3:** Pseudo-first-order rate constants for the aggregation process of PBS-PFP (3.46 × 10^−5^ M), PBS-PFP2 (1.06 × 10^−5^ M when studying the effect of salt and 2.62 × 10^−5^ M when studying the effect of temperature) and PBS-PFP3 (various concentrations indicated in the table). All concentrations are given in terms of CPE repeat units.

		PBS-PFP2						PBS-PFP3			
		Effect of salt— [NaCl] (M)			Effect of temperature (°C)			Effect of concentration (×10^−5^ M)			
Polyelectrolyte	PBS-PFP	0	0.01	0.1	25.5	35.5	45.0	1.63	2.48	3.31	4.98
k (×10^−3^ s^−1^)	0.90	1.4	1.6	1.9	0.90	1.3 ^a^	1.3 ^a^	2.0	1.9	1.7	1.8

^a^ The rate constants at 35.5 and 45.0 °C determined by Guggenheim’s method [58,59] were the same within experimental error.

## Supplementary Materials

The following are available online at http://www.mdpi.com/2073-4360/10/3/258/s1, Figure S1: Normalised fluorescence spectra of aqueous solutions (ca. 50 μM in terms of repeat units) of PBS-PFP (λ_excit_ 370 nm), PBS-PFP2 (λ_excit_ 365 nm), and PBS-PFP3 (λ_excit_ 370 nm).
