# Supplementary material for: Effects of Charge Density on Photophysics and Aggregation Behavior of Anionic Fluorene-Arylene Conjugated Polyelectrolytes
Source: Polymers (Basel). 2018 Mar 2;10(3):258. doi: 10.3390/polym10030258 (PMC6414976; doi:10.3390/polym10030258)
Supplement: Supplementary file 1 [file polymers-10-00258-s001.pdf]

Supporting Information for Publication

Effects of Charge Density on Photophysics and Aggregation Behaviour of Anionic Fluorene-arylene Conjugated Polyelectrolytes

Liliana M. Martelo[1,*,#], Sofia M. Fonseca[1], Ana T. Marques[2], Hugh D. Burrows[1,*], Artur J. M. Valente[1], Licínia L. G. Justino[1], Ullrich Scherf[2], Swapna Pradhan[2], Qiu Song[2, §].

[1] Departamento de Química, Universidade de Coimbra, 3004-535 Coimbra (Portugal); liliana.martelo@tecnico.ulisboa.pt (L.M.M.); sfonseca@qui.uc.pt (S.M.F.); burrows@ci.uc.pt (H.D.B.); avalente@ci.uc.pt (A.J.M.V.); liciniaj@ci.uc.pt (L.L.G.J.)

[2] Makromolekulare Chemie, Bergische Universität Wuppertal, DE-42097 Wuppertal (Germany); anatmarques@yahoo.com (A.T.M.); scherf@uni-wuppertal.com (U.S.); swapna_shivasan@yahoo.com (S.P.); squi2010@sinano.ac.com (Q.S.)

Present address:

[#] Centro de Química-Física Molecular, Instituto Superior Técnico, 1049-001 Lisbon, Portugal

§ Printable Electronics Research Center, Suzhou Institute of Nano-Tech and Nano-Bionics, CAS, 215123, Suzhou, P. R. China

* Correspondence: liliana.martelo@tecnico.ulisboa.pt (LMM); Tel.: +351-218-419-259 and burrows@ci.uc.pt (HDB); Tel.: +351-239-852-083

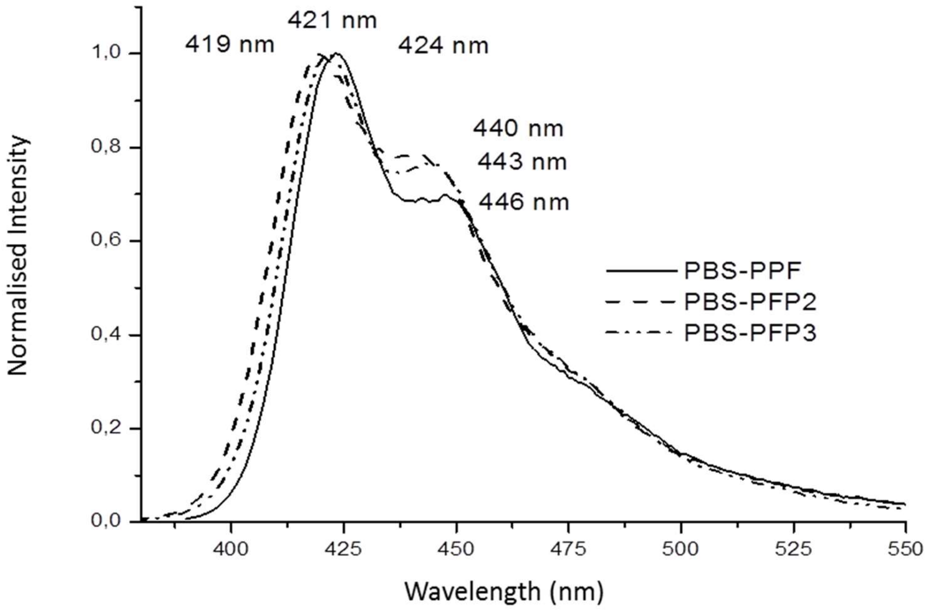

Figure S1 Normalised fluorescence spectra of aqueous solutions (ca. 50 μM in terms of repeat units) of PBS-PFP (λ_{excit} 370 nm), PBS-PFP2 (λ_{excit} 365 nm) and PBS-PFP3 (λ_{excit} 370 nm).
